# Multi-scale problem in the model of RNA virus evolution

**DOI:** 10.1088/1742-6596/727/1/012007

**Published:** 2016-06

**Authors:** Andrei Korobeinikov, Aleksei Archibasov, Vladimir Sobolev

**Affiliations:** Centre de Recerca Matemática, Campus de Bellaterra, Edifici C, 08193 Barcelona, Spainakorobeinikov@crm.cat; Departament de Matemàtiques, Universitat Autònoma de Barcelona, Campus de Bellaterra, Edifici C, 08193 Barcelona, Spainaarchibasov@gmail.com; Department of Applied Mathematics Samara State Aerospace University (SSAU), 34, Moskovskoye shosse, Samara 443086, Russiahsablem@gmail.com; Department of Technical Cybernetics Samara State Aerospace University (SSAU), 34, Moskovskoye shosse, Samara 443086, Russia

## Abstract

A mathematical or computational model in evolutionary biology should necessary combine several comparatively fast processes, which actually drive natural selection and evolution, with a very slow process of evolution. As a result, several very different time scales are simultaneously present in the model; this makes its analytical study an extremely difficult task. However, the significant difference of the time scales implies the existence of a possibility of the model order reduction through a process of time separation. In this paper we conduct the procedure of model order reduction for a reasonably simple model of RNA virus evolution reducing the original system of three integro-partial derivative equations to a single equation. Computations confirm that there is a good fit between the results for the original and reduced models.

## 1. Introduction

Due to very high mutation and replication rates combined with high recombination abilities, RNA viruses are able to evolve very fast. This high evolvability makes RNA viruses a convenient experimental model in evolutionary biology and, at the same time, presents a serious problem, as viral evolution is probably the most important single factor accountable for the emergence of new pathogens and the development of drug resistance by existing ones.

Despite its apparent relevance, so far the development of mathematical or computational models of viral evolution which can be used in combination with experimental studies in evolutionary biology, remains a challenge even for such a simple object as RNA virus. In order to be useful in biological research, a mathematical model should be based on explicitly stated basic principles, postulates and hypotheses and allow a transparent interpretation of both results and parameters. In other words, a useful model should be essentially mechanistic. This implies that in evolutionary biology such a model must include a combination of factors responsible for natural selection (which drives evolution) and a mechanism for describing evolution. However, the factors generating the selection pressure act on a time scale comparable with an average generation time, or a life span of a single entity, whereas evolution is a comparatively slow process of accumulation of small changes that progresses through many generations to be evident. As

Content from this work may be used under the terms of the Creative Commons Attribution 3.0 licence. Any further distribution of this work must maintain attribution to the author(s) and the title of the work, journal citation and DOI.
Published under licence by IOP Publishing Ltd a result, the model unavoidably includes multiple time scales which differ by several orders of magnitude. Computational or analytical handling of such models usually is a highly non-trivial task. In the case of viral evolution this discrepancy is further complicated by the fact that a typical generation times of virus and the hosts differ by orders of magnitude as well.

Besides phenomenological models, such as models suggested by Tsimring, Levin and Kessler [1] or Sasaki [2] and Sasaki and Haraguchi [3, 4], are free of the mentioned drawbacks. However, the use of phenomenological models as a compliment to experimental research is usually questionable, as such models are rather illustrative than explanatory, and interpretation of models' parameters and obtained results in connection with an experimental layout is problematic.

These arguments imply that an ideal model should be mechanistic, include a number of properties of a real–life system and, at the same time, it should be sufficiently simple to allow analysis.

A relatively simple mathematical model of RNA virus evolution was recently suggested by Korobeinikov and Dempsey [5]. This mechanistic model is an extension of Nowak–May HIV model [6], where viral phenotypes (strains) are assumed to be distributed in a continuous phenotype space, and random mutations are described by dispersion. For the sake of simplicity, Korobeinikov and Dempsey formulated the model on a basis of the 2–dimensional Wodarz–Christensen–Thomsen, or Wodarz, model [7]. The latter is a reduction of the original 3–dimensional Nowak–May model under an assumption that the population of free virus particles is proportional to the infected cells population. However, an analytical study of even this comparatively simple model still is a highly challenging task, and it can be expected that for a more complex model, which includes factors, such as immune response or a therapy, the analysis would be even more difficult.

As we mentioned above, to a large extent the model complexity arises as a result of the presence of multiple processes progressing at very different time scales. Thus, the original Nowak–May model combines three processes and three time scales, namely (i) the proliferation of the uninfected target cells, (ii) the infectious process and infected cells' life cycle, and, finally, (iii) free virus life cycle. In the Wodarz model, which was used as a basis for model in [5], one of these processes, namely the free virus life cycle, is omitted and replaced by the assumption that the free virus population is proportional to the infected cells population. The latter makes the model slightly simpler. However, model in [5] includes evolution which occurs on a comparatively slow timescale. Accordingly, the model is a slow–fast system.

The considerable differences of the timescales, which make the model difficult for both computer simulation and analysis, at the same time, indicate that the model can be simplified using the process known as time scales separation (for instance, see [8, 9, 10]). In this paper we carry out such a scale separation. As a result, the model RNA virus evolution in [5] is reduced to a single equation. Simulations demonstrate that apart from a comparatively short transition period there is a good fit of results for both, the full and the reduced models.

We would like to stress that the result is not limited by the specific model introduced in [5]. As the presence of multiple time scales which differ by a several orders of magnitude is typical for mechanistic models in evolutionary biology, the same technique can be applied to any such model.

## 2. The model

As a basis for modelling, we employ the Nowak–May model of HIV dynamics [6]. This model describes interactions of three populations, namely uninfected target cells (for HIV, the target cells are T helper, or Th cells), infected target cells, and free virus particles, of concentrations $u(t)$, $V(t)$ and $X(t)$, respectively. The model postulates that there is a continuous influx of the target cells (from the thymus, where they mature) at a rate $b$, that free virus particles infect target cells at a rate $\alpha X u$, that the infected cells produce free virus particles at a per capita rate $k$, and that average life spans of the uninfected cells, infected cells and the free virus particles are $1/q$, $1/m$ and $1/c$, respectively. Under these assumptions the model equations are

$$\begin{aligned} \frac{du(t)}{dt} &= b - \alpha\, X(t)u(t) - q\, u(t), \\ \frac{dV(t)}{dt} &= \alpha\, X(t)u(t) - m\, V(t), \\ \frac{dX(t)}{dt} &= k\, V(t) - c\, X(t). \end{aligned} \tag{1}$$

The global qualitative behavior of this model is well studied and is known to be completely determined by the basic reproduction number of the virus (or that of infected cells, as for this model these numbers coincide) $R_0 = \alpha bk/cmq$ [11, 12, 13, 14].

Let us assume now that multiple viral strains are possible and are distributed in a continuous phenotype space. For model (1) a viral variant is characterized by parameters $\alpha, k, c$ and $m$, and hence it could be assumed that the phenotype space is 4–dimensional. However, it is known (and we will demonstrate this further) that for the Nowak-May model (1) these parameters are not independent: as we mentioned, for this model the viral dynamics is completely characterized by the basic reproduction number $R_0$ which usually serves as a measure of the Darwinian fitness of the virus. In other words, in the framework of the Nowak-May model a viral phenotype is uniquely characterized by the single number $R_0$, and, therefore, it suffices to consider a 1–dimensional phenotype space $M = \{s \in (0, \infty)\}$. Then fitness $R_0$ is a function of variable $s$. (The graph of this function is usually referred to as a "fitness landscape" .) The variables $v(t, s)$ and $x(t, s)$ are strain-specific and hence are the density distributions of the infected cells population and the virus in the phenotype space, whereas the total infected cells population and the total viral load are $V(t) = \int_0^\infty v(t, s)ds$ and $X(t) = \int_0^\infty x(t, s)ds$, respectively. New strains emerge as a result of random mutations which in a continuous phenotype space can be modelled by dispersion.

In the Nowak–May model coefficients $\alpha$, $m$, $k$ and $c$ are attributed to a phenotype, and hence these are function of $s$. We assume that $\alpha = \alpha(s) = \alpha^0\alpha_0(s)$, $m = m(s) = m^0 m_0(s)$, $k = k(s) = k^0 k_0(s)$ and $c = c(s) = c^0 c_0(s)$, where $\alpha^0$ ,$m^0$, $k^0$ and $c^0$ are coefficients of a wild strain (or a phenotype at the beginning of evolution), $\alpha_0(s)$, $m_0(s)$, $k_0(s)$ and $c_0(s)$ are non–dimensional functions and $m_0(s), k_0(s), c_0(s) = 1$ for this wild phenotype. These assumptions lead to the following system of equations:

$$\begin{aligned} \frac{du(t)}{dt} &= b - u(t) \int_0^\infty \alpha(s)\, x(t, s)\, ds - q\, u(t), \\ \frac{\partial v(t, s)}{\partial t} &= -m(s)\, v(t, s) + \alpha(s)\, u(t)\, x(t, s) + \mu\, \frac{\partial^2 v(t, s)}{\partial s^2}, \\ \frac{\partial x(t, s)}{\partial t} &= k(s)\, v(t, s) - c(s)\, x(t, s). \end{aligned} \tag{2}$$

Note that coefficients $b$ and $q$ are characteristics of target cells, and hence these remain constant.

This system of equations should be complemented by initial and boundary conditions. At $s = +\infty$ the natural boundary conditions for $v(t, s)$ and $x(t, s)$ are zeros. At $s = 0$ for convenience we use no-flux conditions $\frac{\partial v(t, 0)}{\partial s} = 0$. Initial conditions are $u(0) = u^0$, $v(0, s) = v^0(s)$, $x(0, s) = x^0(s)$, where $u^0, v^0(s), x^0(s) > 0$. Here time $t$ is measured in days and variable $s$ is non–dimensional. Furthermore, $u(t)$ is measured in $cells \cdot mm^{-3}$; $v(t,s)$ and $x(t,s)$ are the densities of cells and free virus particles in the phenotype space, and hence these are measured in $cells \cdot mm^{-3}$ and $virions \cdot mm^{-3}$; cell production rate $b$ is measured in $cells \cdot mm^{-3} \cdot day^{-1}$; per capita mortality rates $q$, $m$, $k$ and $c$ are measured in $day^{-1}$, infectivity $\alpha$ is in $mm^3 \cdot virions^{-1} \cdot day^{-1}$, and dispersion coefficient $\mu$ is in $day^{-1}$.

A model of such type (based on the 2–dimensional Wodarz model, which is, in turn, a reduction of the 3–dimensional Nowak–May model) was introduced in [5], and simulations with this model revealed a number of practically relevant outcomes. In particular, the model demonstrates the behavior which closely resembles the observed development of HIV infection. However, an apparent drawback of this model is that even in its present form its analytical study is a challenging task. A further development of the model, which could involve an incorporation of factors such as immune response and a therapy, would make the model more complicated and its analysis even more difficult. The complexity of this model is a result of an interaction of multiple processes with very different time scales. Thus, the original Nowak–May model (1) combines three processes and three time scales, namely (i) the proliferation of uninfected target cells, (ii) the process of infection and infected cells' life cycle, and, finally, (iii) the free virus particles life cycle. Compared with the Nowak–May model, model (2) includes the process of evolution, which, in comparison, is a very slow process, and hence model (2) is a slow-fast system. The presence of four considerably different timescales indicates that model (2) can be significantly simplified if we separate the timescales.

## 3. Timescales separation

Let us denote $t = T\bar{t}, s = S\bar{s}$ and

$$u\left(T\bar{t}\right) = U\bar{u}(\bar{t}), \quad v(T\bar{t}, S\bar{s}) = \tilde{V}\bar{v}\left(\bar{t}, \bar{s}\right), \quad x(T\bar{t}, S\bar{s}) = \tilde{X}\bar{x}\left(\bar{t}, \bar{s}\right),$$

and assume that

$$T = \frac{1}{\mu}, \quad S = 1, \quad U = \frac{b}{q}, \quad \tilde{X} = \frac{k^0}{c^0}\tilde{V}, \quad \tilde{V} = 1\ (cells/s)\,.$$

Substituting these into system (2), we obtain a non–dimensional system

$$\varepsilon\frac{d\bar{u}}{d\bar{t}} = 1 - \int_0^\infty \frac{1}{q}\alpha(\bar{s})\frac{k^0}{c^0}\bar{u}\bar{x}d\bar{s} - \bar{u}, \tag{3}$$

$$\frac{\partial \bar{v}}{\partial \bar{t}} = \alpha(\bar{s})\frac{bk^0}{\mu q c^0}\bar{u}\bar{x} - \frac{m(\bar{s})}{\mu}\bar{v} + \frac{\partial^2 \bar{v}}{\partial \bar{s}^2}, \tag{4}$$

$$\varepsilon\nu\frac{\partial \bar{x}}{\partial \bar{t}} = \bar{k}(\bar{s})\bar{v} - \bar{c}(\bar{s})\bar{x}, \tag{5}$$

where $\varepsilon = \mu/q$ and $\nu = q/c^0$. Initial and boundary conditions for this system are, respectively,

$$\bar{u}(0) = \frac{q}{b}u(0), \quad \bar{v}\left(0, \bar{s}\right) = \frac{1}{\tilde{V}}v(0,s), \quad \bar{x}\left(0, \bar{s}\right) = \frac{1}{\tilde{X}}x(0,s)$$

and

$$\frac{\partial \bar{v}(\bar{t},0)}{\partial \bar{s}} = 0, \quad \lim_{\bar{s}\to\infty}\bar{v}(\bar{t},\bar{s}) = 0.$$

Both numbers, $\varepsilon = \mu/q$ and $\nu = q/c^0$, are small parameters; for instance, for parameters, which were used in [5] and which correspond to a real life HIV case from [15], $\nu \approx 2 \cdot 10^{-3}$ and

$\varepsilon$ ranges from $5 \cdot 10^{-5}$ to $5 \cdot 10^{-3}$. That is, system (3)–(5) is a singularly perturbed ("slow-fast") system, and its size can be reduced using the scale separation technique [8]. This procedure corresponds to assumptions that (i) there is a stable slow manifold such that all solutions converge to this manifold, and hence (ii) the values of fast variables $u(t)$ and $x(t, s)$ converge to quasi-equilibrium values which slowly vary as the system progresses along the slow manifold [9].

Let $\nu = 0$, then equality

$$\bar{k}(\bar{s})\bar{v} = \bar{c}(\bar{s})\bar{x}$$

holds. Substituting this equality into (3) and (4), we obtain equations

$$\varepsilon \frac{d\bar{u}}{d\bar{t}} = 1 - \int_0^\infty \frac{1}{q}\beta(\bar{s})\bar{u}\bar{v}d\bar{s} - \bar{u}, \tag{6}$$

$$\frac{\partial \bar{v}}{\partial \bar{t}} = \frac{b}{\mu q}\beta(\bar{s})\bar{u}\bar{v} - \frac{m(\bar{s})}{\mu}\bar{v} + \frac{\partial^2 \bar{v}}{\partial \bar{s}^2}, \tag{7}$$

where $\beta(\bar{s}) = \alpha(\bar{s})k(\bar{s})/c(\bar{s})$. System (6), (7) was suggested in [5].

Now let $\varepsilon = 0$, then

$$\bar{u} = \frac{1}{1 + \int_0^\infty \frac{1}{q}\beta(\bar{s})\bar{v}d\bar{s}} = \frac{1}{1 + \bar{\lambda}(t)},$$

where $\bar{\lambda}(t) = \int_0^\infty \frac{1}{q}\beta(\bar{s})\bar{v}d\bar{s}$ is the non–dimensional infection force. (Please recall that the dimensional infection force is usually defined as $\lambda(t) = \int_0^\infty \beta(s)v(t,s)ds$.) Substituting this equality into (7), we finally obtain a single equation

$$\frac{\partial \bar{v}}{\partial \bar{t}} = \bar{m}(\bar{s}) \left( \frac{R_0(\bar{s})}{1 + \bar{\lambda}} - 1 \right) \bar{v} + \frac{\partial^2 \bar{v}}{\partial \bar{s}^2}, \tag{8}$$

where $R_0(\bar{s}) = \dfrac{b\alpha(\bar{s})k(\bar{s})}{qm(\bar{s})c(\bar{s})}$, $\qquad \bar{m}(\bar{s}) = \dfrac{m(\bar{s})}{\mu}$.

When $\lambda(t) \ll q$, that is when the reduction of the uninfected population due to infection is small, equation (8) can be further simplified: in this case we can employ the Taylor expansion and use the approximate equality

$$\frac{1}{1 + \bar{\lambda}(t)} \approx 1 - \bar{\lambda}(t).$$

Then

$$\frac{\partial \bar{v}}{\partial \bar{t}} = \bar{m}(\bar{s}) \left[ R_0(s)(1 - \bar{\lambda}) - 1 \right] \bar{v} + \frac{\partial^2 \bar{v}}{\partial \bar{s}^2}.$$

## 4. Numerical simulations

Following [5], and for the sake of simplicity, we assume that the diversity of viral phenotypes is described by a single parameter $\alpha(s)$ which can be interpreted as the efficiency of a single virus particle in infecting a target cell; the other parameters are assumed to be constant and the same for all genotypes. To some extent this assumption is justified by the fact that for the Nowak–May model genotypes fitness is defined by a single number $R_0 = \frac{\alpha bk}{cmq}$. Under this hypothesis, and taking into consideration that $R_0$ is a function of all six parameters, it should make no difference which of these six parameters is varying in the phenotype space. The choice of $\alpha(s)$ is just a matter of convenience, because the direct dependence of $R_0$ on $\alpha$ simplifies the notation.

Results of numerical analysis are presented at the following figures. (For convenience of comparison, results in these figures are shown for physical dimensional variables.) For these simulations we use the following values for the system parameters: $b = 20\ cells \cdot mm^{-3} \cdot day^{-1}$, $m = 0.8\ day^{-1}$, $q = 0.02\ day^{-1}$, $k = 10^4\ day^{-1}$ and $c = 8\ day^{-1}$. These parameters correspond to patient 2 in [15]. For simplicity we assume a linear landscape, postulating that $\alpha(s) = as$. Here $a = 8 \cdot 10^{-7}\ mm^3 \cdot cells^{-1} \cdot day^{-1}$; this implies that $R_0 = 1.25s$. All simulations are for $\mu = 10^{-6}\ day^{-1}$.

Simulations demonstrate an excellent fit of results obtained for system (6), (7) and equation (8).

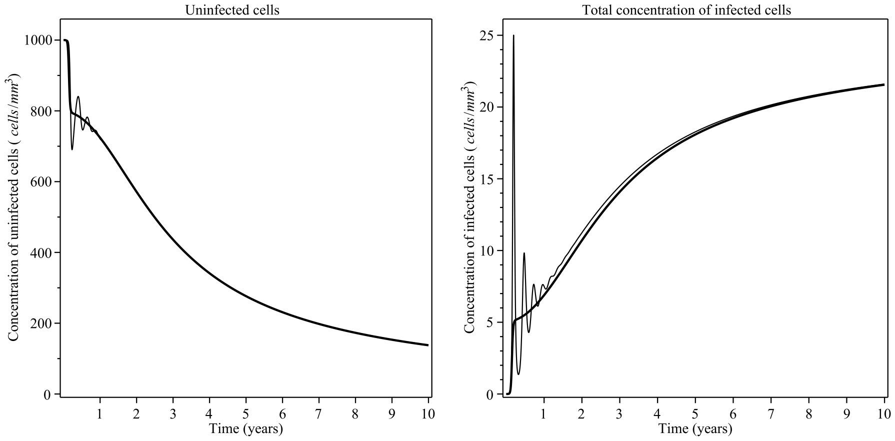

**Figure 1.** Uninfected cells concentration $u(t)$ and total infected cells concentration $V(t)$ for system (6), (7) (thin line) and equation (8) (bold line).

Figure 1 shows that results for these two systems perfectly coincide everywhere apart from a comparatively short transition period. (We have to remind that equation (8) is unable to describe the transition dynamics at all.) If the initial conditions are taken on or near the slow manifold, then the results for these two systems coincide everywhere (see Fig. 2).

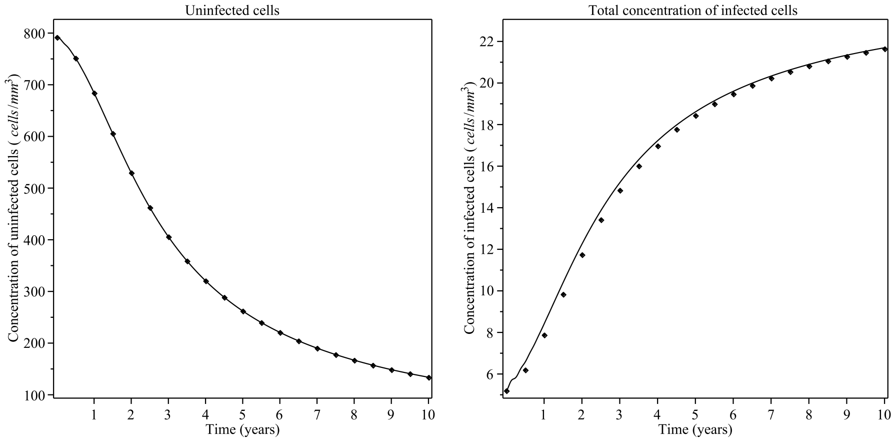

**Figure 2.** Solutions of system (6), (7) (thin line) and equation (8) (markers), when initial condition is taken on the slow manifold.

However, a match of results for systems (3)–(5) and (6), (7) (and hence for equation (8)) is only qualitative. Figure 3 demonstrates that system (3)–(5) exhibits a slower evolution, producing outcomes which are delayed in time, compared with these for system (6), (7), or equation (8).

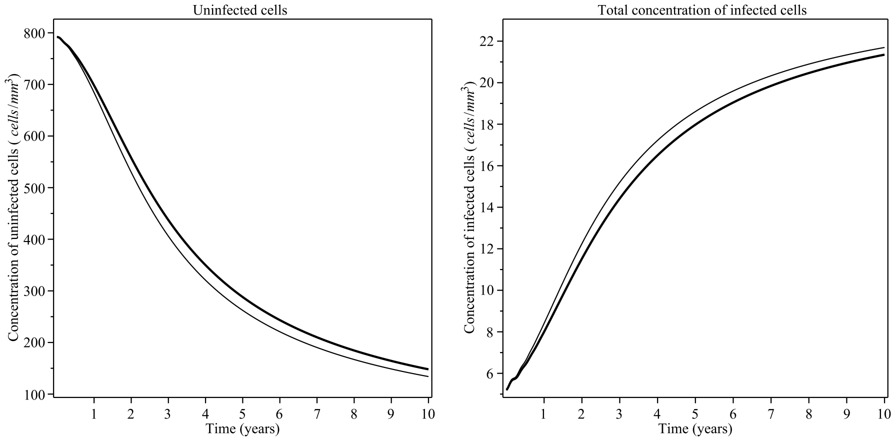

**Figure 3.** Uninfected cells concentration $u(t)$ and total infected cells concentration $V(t)$ for system (3)–(5) (bold line) and (6), (7) (thin line).

We suppose that this effect is caused by a continuous accumulation of a small delay, caused by the third equation of the Nowak–May model (2), over a long period of evolution.

## 5. Summary

Due to the fact that a mathematical model in evolutionary biology should necessary combine the factors, which characterize natural selection and drive evolution, and which acts on a comparatively fast time scale, and a very slow process of evolution itself, such a model is complex and its analysis is difficult. However, a combination of several time scales within a model suggests that such a model can be significantly simplified using the scale separation techniques. For ODE such technique is based on the famous Tikhonov's theorem. The mathematical models in evolutionary biology are usually formulated as integro–differential equations and PDE. However, with some reservations the same concepts can be applied to these equations as well (for instance, see [10, 16, 17]).

In this paper we execute the scale separation for a RNA virus evolution model which is based on the 3–dimensional Nowak–May model. As a result of this procedure, the original system of three integro–partial differential equations is reduced to a single integro–partial differential equation, describing the dynamics of infected target cells $v(t, s)$. The other variables, namely the uninfected cells $u(t)$ and free virus particles $x(t, s)$, are expressed as explicit functions of variable $v(t, s)$. Numerical simulations show an acceptable fit of the solution of the original and the reduced models.

A specific feature of this particular model, which is based on the Nowak–May model specifically tailored to describe the HIV dynamics, is that target cells are assumed to be immigrating from the thymus at a constant rate. However, the same concept and the same techniques can be applied to a model of evolution based on any other model of virus dynamics.

## Acknowledgments

A.K. is supported by the Ministry of Science and Innovation of Spain via Ramón y Cajal Fellowship RYC-2011-08061 and grant MTM2011-29342, by CONACYT (Mexico) via grant N219614, and by AGAUR (Generalitat de Catalunya) via grant 2014SGR-1307.
A.A. is supported by the Russian Foundation for Basic Research via grant 14-01-97018-p and the Ministry of education and science of the Russian Federation in the framework of the implementation of Program of increasing the competitiveness of SSAU for 2013–2020 years.
V.S. is supported by the Russian Foundation for Basic Research via grant 13–01–97002-p and the Ministry of education and science of the Russian Federation in the framework of the implementation of Program of increasing the competitiveness of SSAU for 2013–2020 years.

## References

[1] Tsimring L S, Levine H, Kessler D A (1996). RNA virus evolution via a fitness-space model. Phys. Rev. Lett..

[2] Sasaki A (1994). Evolution of antigenic drift/switching: continuously evading pathogens J. Theor. Biol..

[3] Haraguchi Y, Sasaki A (1997). Evolutionary pattern of intra-host pathogen antigenic drift: effect of crossreactivity in immune response. Phil. Trans. R. Soc..

[4] Sasaki A, Haraguchi Y (2000). Antigenic drift of viruses within a host: a finite site model with demographic stochasticity. J. Mol. Evol..

[5] Korobeinikov A, Dempsey C (2014). A continuous phenotype space model of RNA virus evolution within a *host Math*. Biosci. Eng..

[6] Nowak M A, May R M (2000). Virus dynamics: Mathematical principles of Immunology and Virology.

[7] Wodarz D, Christensen J P, Thomsen A R (2002). The importance of lytic and nonlytic immune responses in viral infections. TRENDS in Immunology.

[8] Khalil H K (1987). Stability analysis of nonlinear multiparameter singularly perturbed systems. IEEE Trans. Aut. Control AC.

[9] Mortell M P, O'Malley R E, Pokrovskii A, Sobolev V A (2005). Singular Perturbation and Hysteresis.

[10] Vasilieva A B, Butuzov V F, Kalachev L V (1995). it The Boundary Function Method for Singular Perturbation Problems.

[11] Korobeinikov A (2004). Global properties of basic virus dynamics models. Bull. Math. Biol..

[12] Korobeinikov A (2009). Global asymptotic properties of virus dynamics models with dose dependent parasite reproduction and virulence, and nonlinear incidence rate. Math. Med. Biol..

[13] Korobeinikov A (2009). Stability of ecosystem: Global properties of a general prey-predator model. Math. Med. Biol..

[14] Vargas-De-León C, Korobeinikov A (2013). Global stability of a population dynamics model with inhibition and negative feedback. Math. Med. Biol..

[15] Stafford M A, Corey L, Cao Y, Daar E S, Ho D D, Perlson A S (2000). Modeling plasma virus concentration during primary HIV infection. J. Theor. Biol..

[16] Butuzov V F, Nefedov N N, Recke L, Schnieder K R (2012). Global region of attraction of a periodic solution to a singularly perturbed parabolic problem. Applicable Analysis.

[17] Nefedov N N, Nikitin A G (2007). The Cauchy problem for a singularly perturbed integro-differential Fredholm equation. Computational Mathematics and Mathematical Physics.

